# FRAILTY AND THE EFFECTIVENESS AND SAFETY OF NEW GLUCOSE-LOWERING DRUGS IN OLDER ADULTS WITH TYPE 2 DIABETES

**DOI:** 10.1093/geroni/igz038.2154

**Published:** 2019-11-08

**Authors:** Dae H Kim, Elisabetta Patorno, Medha Munshi

**Affiliations:** 1 Hebrew SeniorLife, Boston, Massachusetts, United States; 2 Brigham and Women’s Hospital and Harvard Medical School, Boston, Massachusetts, United States; 3 Beth Israel Deaconess Medical Center, Boston, Massachusetts, United States

## Abstract

Recently, several classes of non-insulin glucose-lowering drugs, such as dipeptidyl peptidase-4 inhibitors (DPP4i), glucagon-like peptide-1 receptor agonists (GLP1-RA), and sodium glucose co-transporter-2 inhibitors (SGLT2i), have been approved to treat type 2 diabetes. Clinical trials have shown that GLP1RA and SGLT2i confer cardiovascular benefit, while DPP4i do not have such benefit; these drugs do not seem to increase the risk of hypoglycemia. However, due to underrepresentation of older adults with frailty and lack of frailty assessment in clinical trials, little is known about how the effectiveness and safety of these drugs change across different levels of frailty. In this symposium, we present the real-world evidence from Medicare data April 2013-December 2016 on the utilization trends of newly approved diabetes drugs (Dr. Dave) and comparative effectiveness and safety of SGLT2i vs sulfonylurea (Dr. Pawar), SGLT2i vs DPP4i (Dr. Kim), and SGLT2i vs GLP1-RA (Dr. Patorno). The outcomes were 1) composite cardiovascular endpoint of mortality, myocardial infarction, stroke, or heart failure; and 2) severe hypoglycemia, defined as emergency department visits or hospitalizations due to hypoglycemia. We applied a validated claims-based frailty index (CFI) to estimate the treatment effectiveness and safety in non-frail (CFI<0.10), pre-frail (CFI 0.10-0.19), or frail individuals (CFI≥0.20). Following individual presentations, Dr. Munshi and presenters will lead an interactive discussion about clinical implications and methodological challenges in conducting geriatric pharmacoepidemiologic studies using Medicare data. This symposium will demonstrate the utility of CFI in claims-based pharmacoepidemiologic studies and provide health care providers with new evidence to tailor diabetes pharmacotherapy based on frailty.

